# A large cohort of primary familial cryofibrinogenemia originates from the Magdalen Islands

**DOI:** 10.1186/1710-1492-7-S2-A34

**Published:** 2011-11-14

**Authors:** P Bégin, M Picard, J Paradis, L Paradis, G Leclerc

**Affiliations:** 1 Department of medicine, Service of allergy and immunology, Centre Hospitalier de l’Université de Montréal, Montréal, Qc, Canada; 2 Department of medicine, Chicoutimi Hospital, Saguenay, Qc, Canada

## Background

Cryofibrinogenemia is a rare disorder that refers to the presence of cold-precipitable proteins in plasma but, unlike cryoglobulinemia, not in serum. It can manifest as vascular occlusion in cold exposed areas. It is most often secondary to various inflammatory disorders, infections or malignancy, but cases of true essential cryofibrinogenemia have been described. To the best of our knowledge only three reports involving families have been published to date, each involving at most three patients.

## Our cases

Two apparently unrelated patients presented with painful lesions involving cold-exposed areas that would appear every fall and disappear every spring since childhood. Examination revealed ulcers, livedoid and purple-blue discolorations with crusts and scar tissues involving ears, hips, knees, fingers and toes. Immune and inflammatory workup was unremarkable in both patients except for the presence of cryofibrinogen. Treatment with stanozol and dextran was attempted but symptoms returned during winter season.

**Figure 1:**
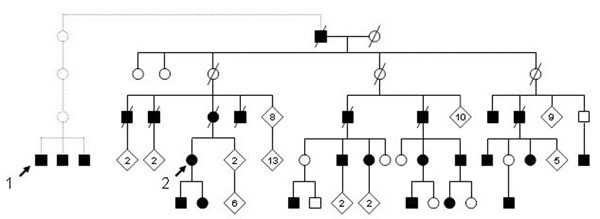
Genetic family tree showing relation between index cases (shown with arrow). Darkened figures indicate subjects reporting symptoms of cryofibrinogenemia.

As both patients reported kindred with similar symptoms, population register was consulted. Patients were found to have common ancestors originating from the Magdalen islands. Furthermore, 24 more individuals from the same family were found to present similar symptoms upon cold exposure, making this the largest cohort of familial cryofibrinogenemia described to date (figure 1). Transmission appeared to follow a dominant pattern with variable penetrance.

## Conclusion

We report a large cohort of familial essential cryofibrinogenemia originating from the Magdalen Islands. Genetic association studies will be necessary to identify the causal gene.
